# Ammonia Emission and Deposition in Scotland and Its Potential Environmental Impacts

**DOI:** 10.1100/tsw.2004.130

**Published:** 2004-09-02

**Authors:** M.A. Sutton, U. Dragosits, S. Hellsten, C.J. Place, A.J. Dore, Y.S. Tang, N. van Dijk, L. Love, N. Fournier, M. Vieno, K.J. Weston, R.I. Smith, M. Coyle, D. Roy, J. Hall, D. Fowler

**Affiliations:** ^1^ Centre for Ecology and Hydrology Edinburgh Research Station, Bush Estate, Penicuik, Midlothian EH26 0QB, UK; ^2^ University of Edinburgh, Department of Geography, Drummond Street, Edinburgh EH8 9XP, UK; ^3^ University of Edinburgh, Institute for Meteorology, Kings Buildings, Edinburgh EH9 3JZ, UK; ^4^ Centre for Ecology and Hydrology, Monks Wood Research Station, Abbots Ripton, Cambridgeshire PE28 2LS, UK

**Keywords:** ammonia, NH_3_, emissions, mapping, models, monitoring network, wet deposition, dry deposition, critical loads, mitigation strategies

## Abstract

The main source of atmospheric ammonia (NH_3_) in Scotland is livestock agriculture, which accounts for 85% of emissions. The local magnitude of emissions therefore depends on livestock density, type, and management, with major differences occurring in various parts of Scotland. Local differences in agricultural activities therefore result in a wide range of NH_3_ emissions, ranging from less than 0.2 kg N ha^−1^ year^−1^ in remote areas of the Scottish Highlands to over 100 kg N ha^−1^ year^−1^ in areas with intensive poultry farming. Scotland can be divided loosely into upland and lowland areas, with NH_3_ emission being less than and more than 5 kg N ha^−1^ year^−1^, respectively.Many semi-natural ecosystems in Scotland are vulnerable to nitrogen deposition, including bogs, moorlands, and the woodland ground flora. Because NH_3_ emissions occur in the rural environment, the local deposition to sensitive ecosystems may be large, making it essential to assess the spatial distribution of NH_3_ emissions and deposition. A spatial model is applied here to map NH_3_ emissions and these estimates are applied in atmospheric dispersion and deposition models to estimate atmospheric concentrations of NH_3_ and NH_4_^+^, dry deposition of NH_3_, and wet deposition of NHx. Although there is a high level of local variability, modelled NH_3_ concentrations show good agreement with the National Ammonia Monitoring Network, while wet deposition is largest at high altitude sites in the south and west of Scotland. Comparison of the modelled NH_x_ deposition fields with estimated thresholds for environmental effects (“critical loads”) shows that thresholds are exceeded across most of lowland Scotland and the Southern Uplands. Only in the cleanest parts of the north and west is nitrogen deposition not a cause for concern. Given that the most intense effects occur within a few kilometres of sources, it is suggested that local spatial abatement policies would be a useful complement to traditional policies that mitigate environmental effects based on emission reduction technologies.

## INTRODUCTION

Ammonia is one of the suite of pollutants that contribute to transboundary air pollution problems, including acid deposition and nitrogen deposition. While sulphur dioxide ($SO_2$) and nitrogen oxides ($NO_x$) produce sulphuric acid and nitric acid in the atmosphere, ammonia is a base and acts as the main neutralizing compound for these species. This leads to the production of ammonium sulphates and ammonium nitrate, and it is in this form that much of the long-range atmospheric transport takes place. Fig. 1 provides a schematic view of the major sources and fate of these pollutants[1]. Whereas $SO_2$ and $NO_x$ emissions result primarily from combustion sources, most $NH_3$ emission derives from agricultural activities[2,3,4]. Most of the primary pollutant gases are either removed by dry deposition directly to the surface of the earth or they react to form ammonium sulphate and nitrate salts, which are present in submicron aerosols (or particles) in the atmosphere. Most of these aerosols are removed by the scavenging action of cloud and rain droplets and are deposited to earth in precipitation. Much more attention has been given to $SO_2$ and $NO_x$ emissions in recent years, and by comparison both emissions and deposition estimates of $NH_3$ are much more uncertain. This uncertainty is important as both $SO_2$ and $NO_x$ emissions are now substantially decreasing following emission abatement policies. Conversely, $NH_3$ emissions have only decreased very slightly over the last 10 years and now represent a much-increased fraction of the total emission of potentially acidifying species[1,5].

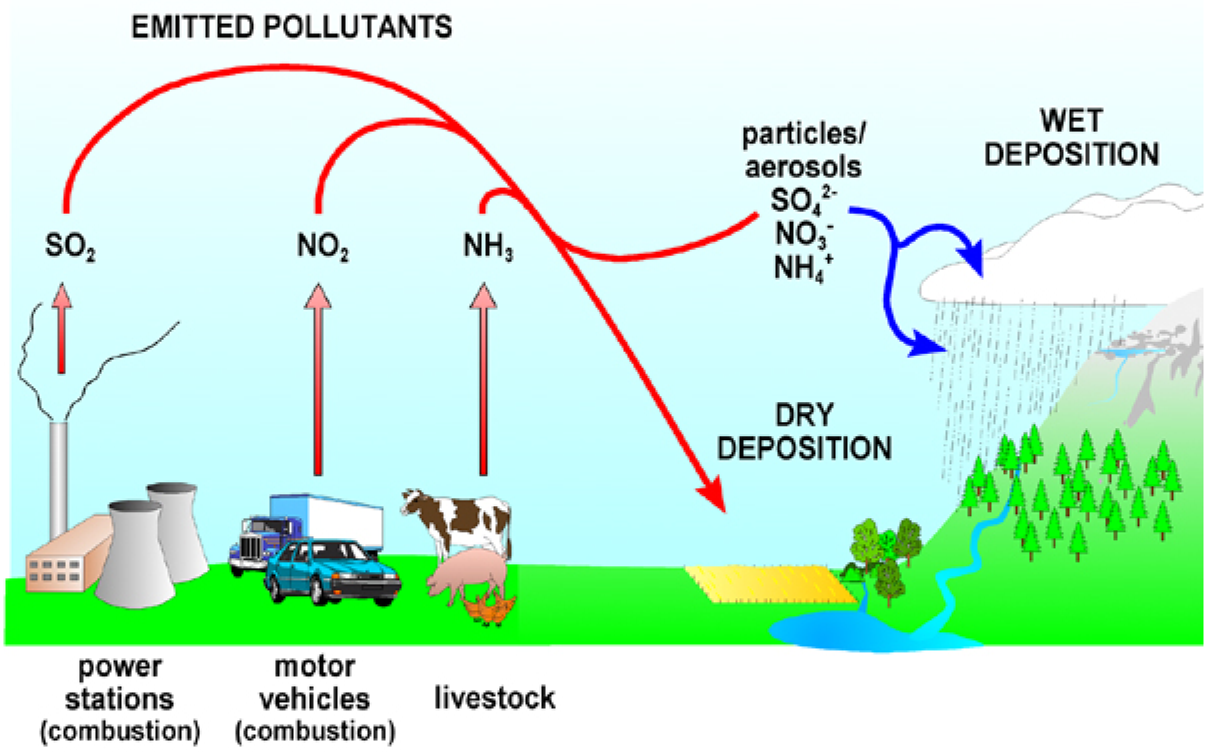

**FIGURE 1.** Schematic of the major sources and pathways of $NH_3$, $SO_2$, and $NO_x$ in the atmosphere (after [1]).

It may appear confusing that $NH_3$ is noted as an "acidifying pollutant" when it is itself a base. The reason for this is that deposited $NH_3$ and $NH_4^+$ (collectively $NH_x$) may be oxidized to nitrate in the soil, a process that is acidifying. Fig. 2 shows that the amount of acidity generated from $NH_x$ depends on the form in which it is deposited as well as its fate. Where $NH_3$ is deposited and accumulated in organic matter, it does not have an acidifying effect[1,6]. Conversely, it should be noted that most $SO_4^{2-}$ and $NO_3^-$ deposited to ecosystems occurs in the form of $NH_4^+$ salts rather than as the un-neutralized acids. The result is that most of the potential acid deposition to ecosystems arrives in the form of $NH_3$ or $NH_4^+$, with $NH_4^+$ acting as the vector for acidity from $SO_2$ and $NO_x$ emissions.

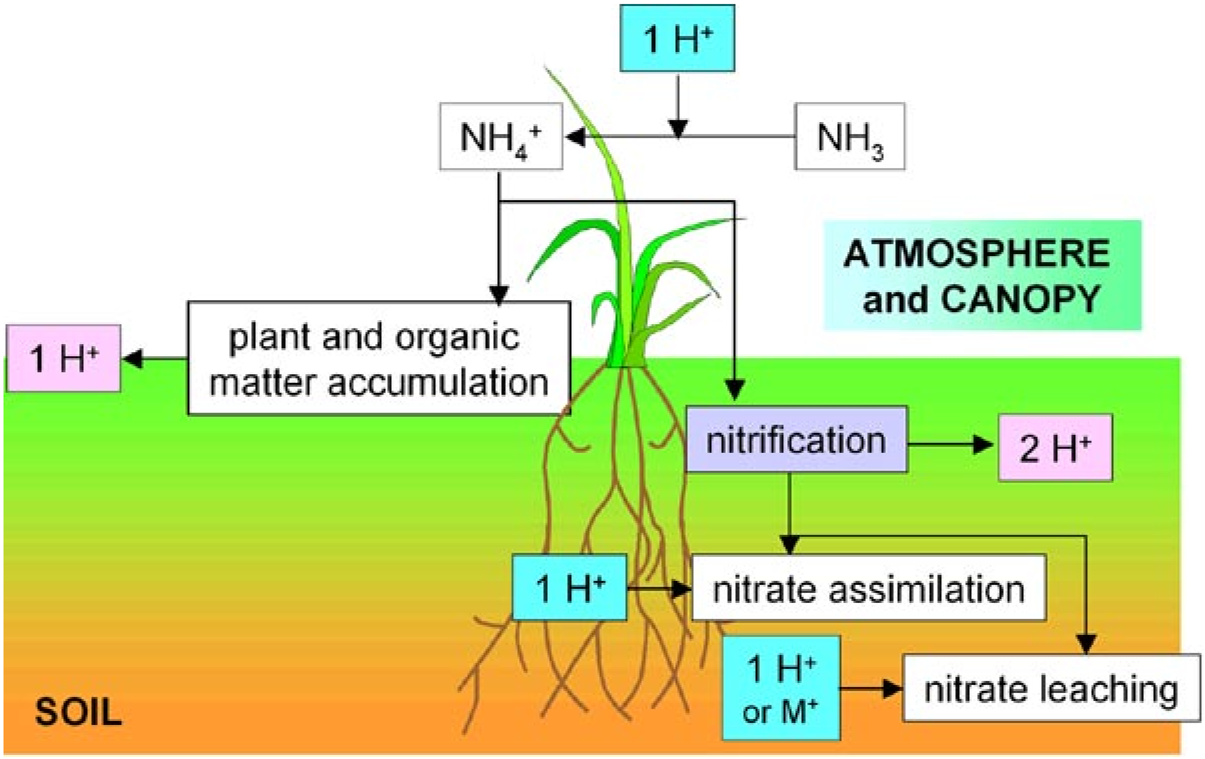

**FIGURE 2.** Fate of $NH_3$ and $NH_4^+$ in plant and soil systems in relation to potential soil acidification (after [1,6]).

Another consequence of $NH_x$ deposition is the supply of additional nitrogen to ecosystems. While the inputs are small compared with agricultural inputs to intensively managed agricultural systems, atmospheric deposition can have a major influence on the nutrient cycling and species composition of semi-natural habitats. Ecosystems adapted to low nutrient availability, such as bogs, moorlands, and mountain ecosystems, are particularly vulnerable, while major changes have also been observed for the ground flora of woodland ecosystems (e.g., [7, 8]).

Ammonia also contributes to a wide range of other environmental impacts. For example, deposition of $NH_x$ leads to additional soil emissions of the greenhouse gas nitrous oxide ($N_2O$)[9]. By contrast, the aerosol ammonium sulphates and nitrate produced by $NH_3$ emissions are thought to lead to a negative effect on the greenhouse balance[10]. While there is much uncertainty over the complex set of interactions involved, in simple terms the effect can be considered as due to both the direct effect of increased light scattering by the ammonium sulphate/nitrate components and the indirect effect of these aerosol components on global reflectance by acting as cloud condensation nuclei. At present, it is unclear whether the $N_2O$ or aerosol effect is larger and whether $NH_3$ emissions have a net positive or net negative effect on the global radiative balance. Finally, it may be noted that the $NH_4^+$ aerosols reduce atmospheric visibility[11] and also contribute to impacts of aerosols on human health[12,13].

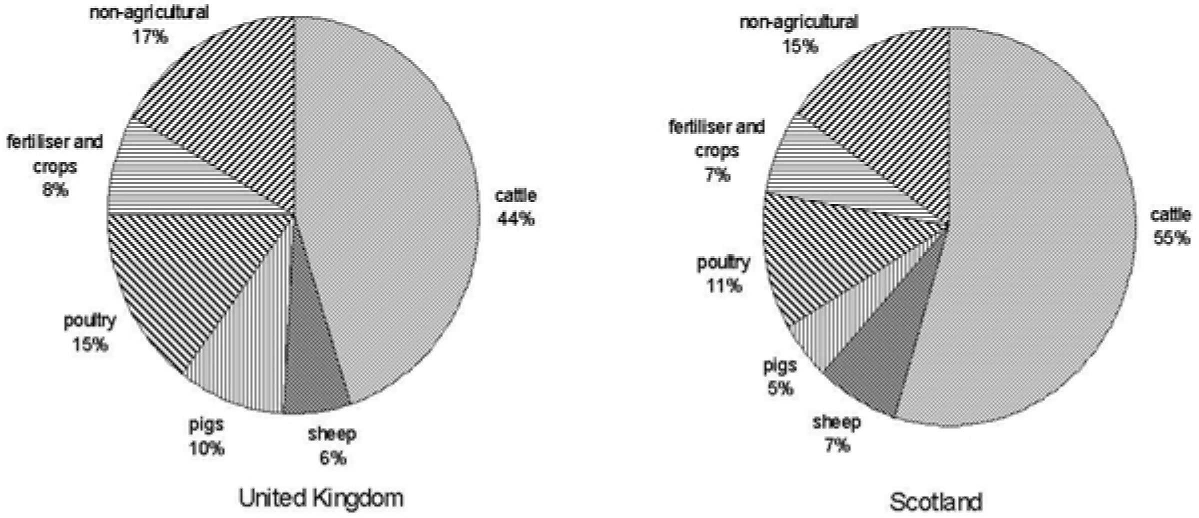

**FIGURE 3.** Sources of ammonia emissions in the U.K. and Scotland (for 1996, other recent years are similar)[2,4].

While Scotland is often thought of as a country with clean air, it is clear that it has large areas with intensive agricultural activity. As a result, there are significant emissions, air concentrations, and deposition of $NH_x$. As shown in Fig. 3, around 85% of ammonia emissions in Scotland result from agricultural activities. This is similar to the U.K. as a whole, although in Scotland the proportion of emissions from cattle (55%) is larger than for the U.K. average (Fig. 3). At 11%, the poultry industry is the second largest source of $NH_3$ emissions in Scotland. A large number of minor sources contribute to the non-agricultural $NH_3$ emissions and these include vehicles fitted with catalytic converters, industrial sources, pets, horses, wild animals, and even people themselves[4]. Of the total U.K. $NH_3$ emission of 285 kt N year$^{-1}$(or Gg N year$^{-1}$), approximately 40 kt N year$^{-1}$ occurs in Scotland. Although the average emission density of $NH_3$ is lower in Scotland than across the U.K., the $NH_3$ emission per head of human population is around 1.7 times the U.K. average.

In order to assess the impacts of atmospheric ammonia emissions on ecosystems, it is essential to be able to model the location of emissions and use the model output to assess the patterns of concentration and deposition. In this paper, we report the application of a GIS $NH_3$ emission model (AENEID, Ammonia Emissions for National Environmental Impacts Determination) for Scotland, which is used to provide input to the FRAME (Fine Resolution Atmospheric Multi-pollutant Exchange) atmospheric dispersion model. The results are compared with the findings of the National Ammonia Monitoring Network and used as input to model the spatial pattern of $NH_x$ deposition. A comparison with deposition thresholds for environmental impacts (critical loads) is also used to show areas where environmental impacts of $NH_x$ on ecosystems are expected across Scotland.

## METHODS

### Ammonia Emissions

Ammonia emissions were mapped using the emission estimates from [2,4]. In the case of agricultural sources, the national values were decomposed spatially using estimates of emission per unit livestock or area of crop for each main sector (cattle, pigs, sheep, poultry, winter wheat, barley, oats, etc.). This assumes, for example, the emission from an average pig or hectare of oats is the same in all parts of Scotland and takes no account of the effect of climatic and soil variations.

Agricultural statistics were available at a parish level from annual census information and a methodology was devised to distribute these sources as ammonia emissions at a 1-km grid resolution. In an earlier modelling approach as used by Sutton et al.[14] and Eager[3], a general method had been used to distribute the sources (see Fig. 4, "Old Method"). In that old approach, the sources in a parish (e.g., beef cattle) were distributed onto possible 1-km grid squares depending on the dominant land cover of each square. It was there assumed, for example, that beef cattle would not be present in a 1-km square dominated by forest. Errors in that approach were minimized by averaging up to a 5-km level. It may be noted, however, that old method distributed the sources as sources (e.g., livestock numbers) and only specified where they cannot occur.

Dragosits et al.[15] developed a new model approach to map ammonia emissions that is used in the present study. This (a) distributes the sources in terms of the likely occurrence of ammonia emissions, (b) gives a positive weighting of likelihood, and (c) allows for each 1-km square to contain different land cover types. In this methodology (referred to as the AENEID model), the component emissions for each source (animal housing, manure storage, field spreading of manures, and grazing) are allocated with a weighting to different land cover types, generalized from the satellite-based CEH land cover data. Depending on the proportion of ammonia emission from each source occurring at each of these four stages, a positive weighting is derived. The results of the new methodology used here in an area of northeast Scotland can be seen in Fig. 4 ("Revised Method"). Comparison with the old method shows major differences, with the emissions concentrated in the valley areas where most good grassland and arable land are present. The difference is particularly clear in the southeast sector of the map (Upper Speyside).

A similar new methodology was developed in the AENEID model for emissions from fertilizers and arable crops[15]. In the case of non-agricultural sources, a wide range of approaches was used and it is recognized that these sources are among the most uncertain both in terms of absolute value and spatial distribution. For many sources, the distribution was linked to human population density at a 10-km level, while seabird emissions were linked to the occurrence of coastal cliff areas. Overall, the new methodology significantly improves the quality of the ammonia emission estimates by better locating emissions in relation to land suitability at a 1-km level and by a more comprehensive inclusion of different minor source types. The new methodology has been applied for the years 1988, 1996, and 1999.

## Ammonia Concentrations and Deposition

Ammonia emissions from the AENEID model provide a key input to model the distribution of ammonia concentrations and deposition, which was conducted by the FRAME model[16,17]. FRAME is a statistical Lagrangian model that operates using straight-line trajectories and a multilayer approach with 33 layers. This allows for an explicit description of vertical dispersion from surface $NH_3$ sources, allowing a better description of $NH_3$ concentrations near ground level than would be possible using a single layer model. A further key feature of FRAME is that it distinguishes between different land cover types for the purpose of estimating dry deposition. These are forest, low semi-natural vegetation, arable land, agricultural grassland, and urban areas. For each land cover type, a different dry deposition velocity is set, based on characteristic values of the canopy resistance for the land cover type in question and locally varing surface windspeeds, as these affect the atmospheric and quasi-laminar boundary layer resistances. The different resistance values set result in larger deposition rates to forests and low semi-natural vegetation than to agricultural land, which is by contrast frequently a net source of ammonia to the atmosphere. FRAME uses emissions of $NH_3$, $SO_2$, and $NO_x$ to allow for reactions between these species in the model atmosphere, and in this study FRAME applied emissions for 1996. The AENEID model provided the $NH_3$ emission estimates, while $SO_2$ and $NO_x$ emissions were provided from the U.K. National Atmospheric Emission Inventory[18].

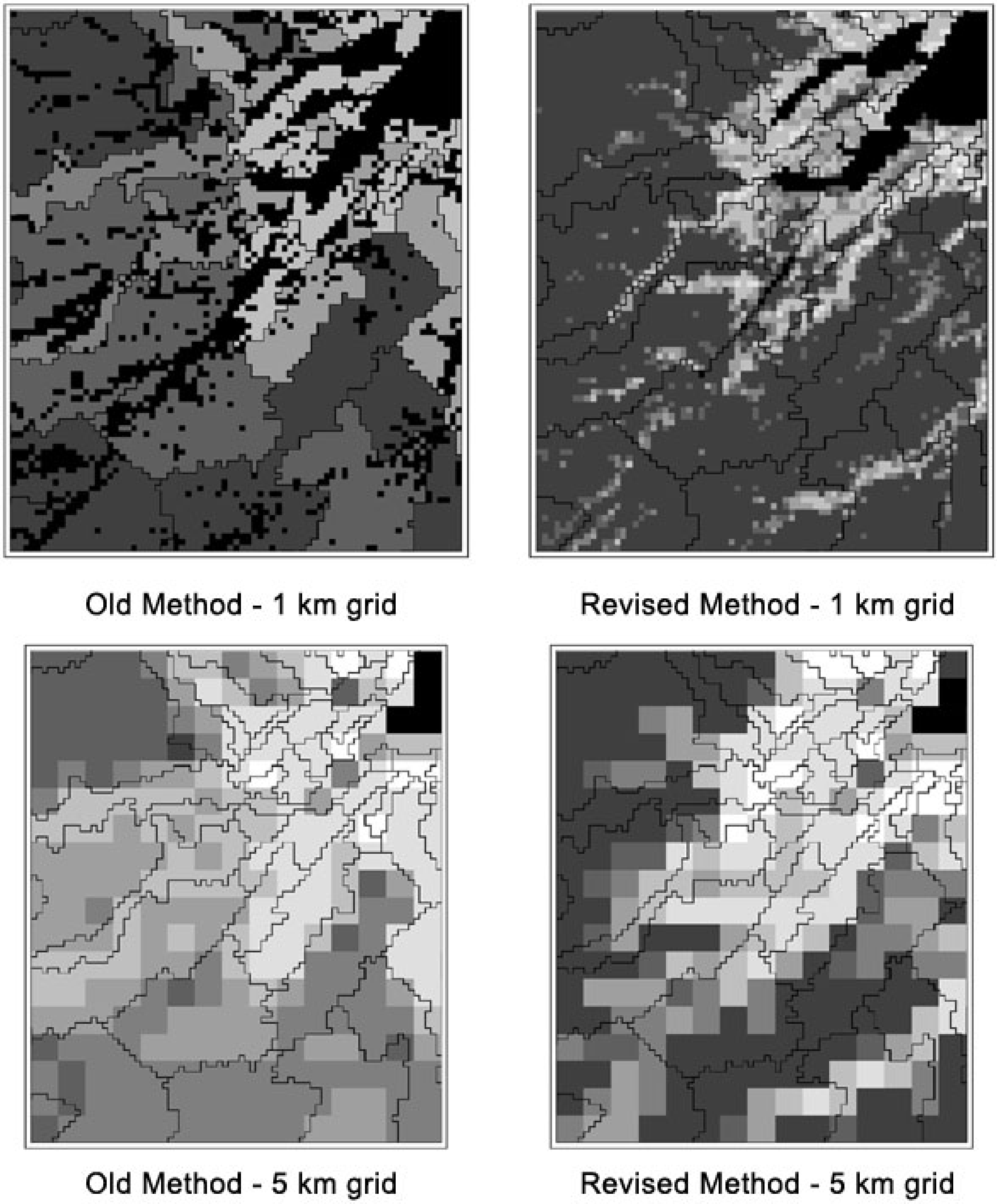

**FIGURE 4.** Comparison of old and new model approaches for distributing ammonia emissions at 1- and 5-km grid resolution. The maps show ammonia emissions from beef cattle around the Black Isle (Lat. 57.6; Long. –4.3), Great Glen (Lat. 57.4, Long. –4.2 to Lat. 57.1, Long. –4.7), Upper Speyside (Lat. 57.3; Long. –3.6), and surrounding areas for 1988. White indicates a high ammonia emission and black a low ammonia emission. The irregular boundaries show parishes mapped on a 1-km grid. For details of the methodology see text.

It is of interest to evaluate the FRAME model estimates of $NH_3$ and $NH_4^+$ concentrations and wet and dry deposition by comparison with measurements. Recognizing this need, the National Ammonia Monitoring Network[19] was established to monitor the spatial patterns and long-term trends of both gaseous $NH_3$ and aerosol $NH_4^+$. Sampling methods were evaluated for reliable sampling on a monthly basis, at a cost permitting sampling at 50–100 sites across the U.K.[20], and led to the development of the CEH DELTA system (DEnuder for Long-Term Ammonia)[21]. This method is applied to sample both $NH_3$ and $NH_4^+$ concentrations, while an improved passive method, the CEH ALPHA (Adapted Low-cost, Passive High-Absorption) sampler[22] is applied at selected sites to measure $NH_3$ only where active sampling is not possible.

In order to provide the best estimates of ammonia wet and dry deposition for Scotland, a combination of measurement and modelling approaches is used. For wet deposition, results from the National Atmospheric Deposition Monitoring Network are used, together with a correction to account for the altitude dependence of wet deposition[1]. For dry deposition of $NO_y$, the monitored air concentration field is interpolated and applied in an inferential model using calculated deposition velocities[23]. In the case of $NH_3$ dry deposition, the FRAME $NH_3$ concentration estimates are refined by an overall calibration against the measurements conducted at a U.K. level[19] and then used as input into the inferential dry deposition model of Smith et al.[23]. As with FRAME, this model accounts for the fact that the rates of dry deposition differ according to land cover type.

## Potential Impacts of Nitrogen Deposition

The "critical loads" methodology is now well established as a tool to estimate the distribution of potential environmental impacts due to atmospheric nitrogen deposition. Critical loads for nitrogen have been set at the European level and provide "*a quantitative estimate of an exposure to deposition of nitrogen as* $NH_x$ *and/or* $NO_y$*, below which harmful effects in ecosystem structure and function do not occur according to present knowledge*"[24], where $NO_y$ refers to the sum of all reactive oxidized nitrogen species (excluding $N_2O$) and, as noted previously, $NH_x$ refers to the sum of all deposited reduced nitrogen. A wide range of methodologies has been established to estimate critical loads for nitrogen deposition, including methods to estimate the effects of acidification and eutrophication by empirical estimates as well as by steady-state and dynamic models[25]. In most methods, a fundamental assumption is made that the effects of different forms of nitrogen deposition (wet vs. dry, oxidized vs. reduced) are the same. While this assumption is for practicality continued in the present estimates to allow N deposition to be considered as a common unit, it is recognized that in practice there are likely to be different effects of the different N forms. For example, $NH_x$ may have larger effects per unit N deposited than $NO_y$ on sensitive cryptogamic plants, while $NO_y$ may have a more direct effect on soil acidification, which remain issues for ongoing research.

A detailed study mapping critical loads for habitats and species for Scotland was reported by Fowler et al.[26] and this approach is applied in the present study. In the case of the eutrophicating effects of nitrogen deposition on species composition, critical loads are most closely linked to habitat type and it is on this basis that the critical loads for nitrogen eutrophication have been estimated. In the case of bog, heathland, and grassland habitats, empirical values of the critical loads were used, based on the results from a range of experimental studies. Conversely, when mapping critical loads for deciduous and coniferous woodland in the U.K., estimates based on the mass balance of nitrogen in the system are also used, which assesses the minimum total nitrogen deposition that would balance against acceptable "near natural" N sinks in the ecosystem (such as forest growth, natural $N_2O$ emission, etc.), so that nitrate leaching from the soil is maintained at natural values[25].

Estimating a critical load for total atmopsheric nitrogen deposition to a particular species is much more difficult, since the species may be associated with a number of habitats. However, in some instances, key species are linked to specific habitats. For example, the sundew (*Drosera rotundifolia*) may be linked, in particular, to bog habitats and heath habitats. In this case, Fowler et al.[26] were able to estimate a critical load of 10 kg N $ha^{-1}$ $year^{-1}$, based on the empirical habitat critical loads for total nitrogen deposition. This value was combined with the potential distribution of *D. rotundifolia*, by using 10-km grid squares where the plant has been recorded either before or after 1950.

## RESULTS

### Ammonia Emissions

The estimated distribution of ammonia emissions in Scotland for 1999 is shown in Fig. 5A. Scotland can be loosely divided into two halves, with less than 5 kg N ha$^{-1}$ year$^{-1}$ emission in upland areas (Highlands and Southern Uplands) and more than 5 kg N ha$^{-1}$ year$^{-1}$ in lowland areas (central and eastern Scotland, plus the southern part of Dumfries and Galloway). Scotland includes some of the highest and lowest $NH_3$ emission densities in the U.K. On the one hand, hot spots of $NH_3$ emission, which exceed 50 kg N ha$^{-1}$ year$^{-1}$, are found in some lowland areas. Conversely, the lowest $NH_3$ emission densities in the northwest Highlands are less than 0.5 kg N ha$^{-1}$ year$^{-1}$.

While Fig. 5A demonstrates the huge spatial variability in $NH_3$ emissions in Scotland, Fig. 5B provides an indication of the main source sector contributions across the country. Because of data disclosivity issues, it is not possible to report this map for the main modelled years of 1996 or 1999, and the picture shown here is for 1988. The main spatial patterns are, however, rather similar between the

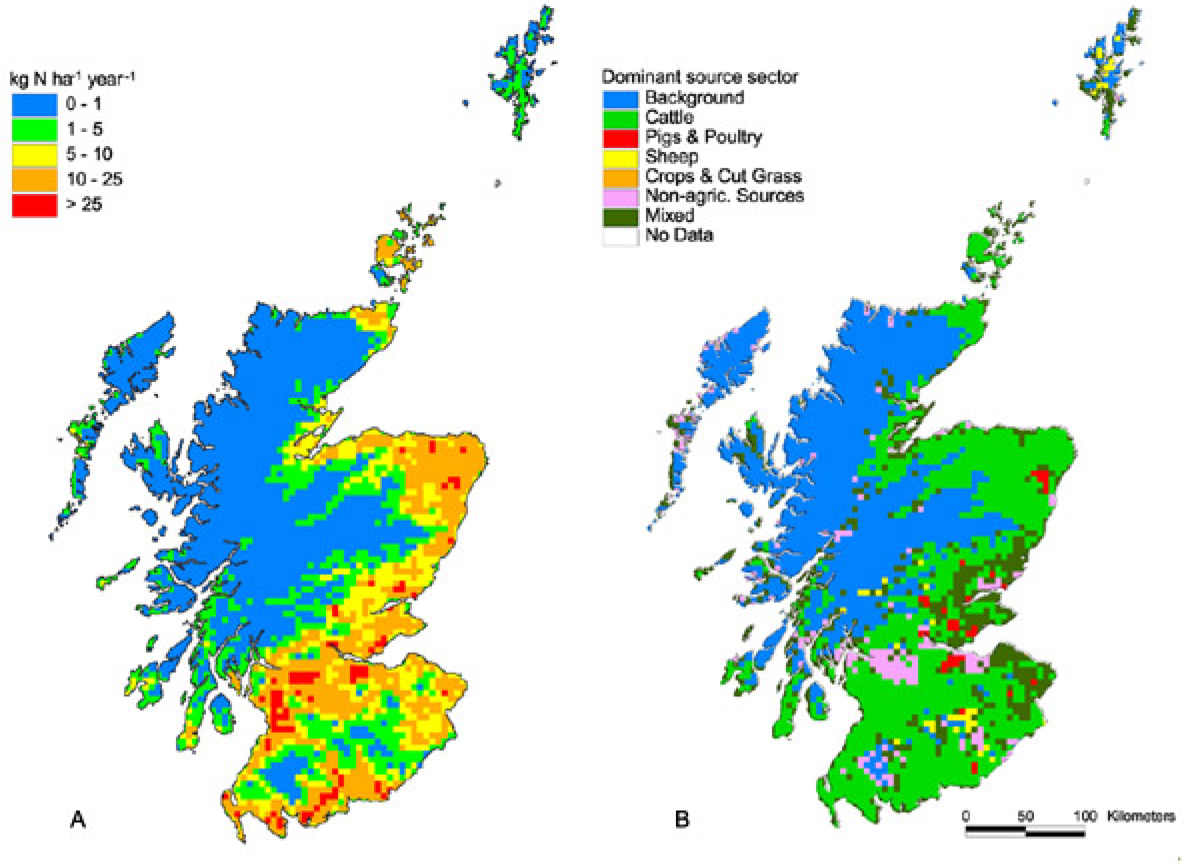

**FIGURE 5.** Estimated distribution of ammonia emissions for 1999 (A, left) and the main contributing sources (B, right) across Scotland (for 1988; see text for explanation of different year). In map B, “background” indicates that emissions are less than 1 kg N ha$^{-1}$ year$^{-1}$ averaged over a 5-km grid square. Mixed indicates that no single sector provides ≥45% of the total $NH_3$ emission.

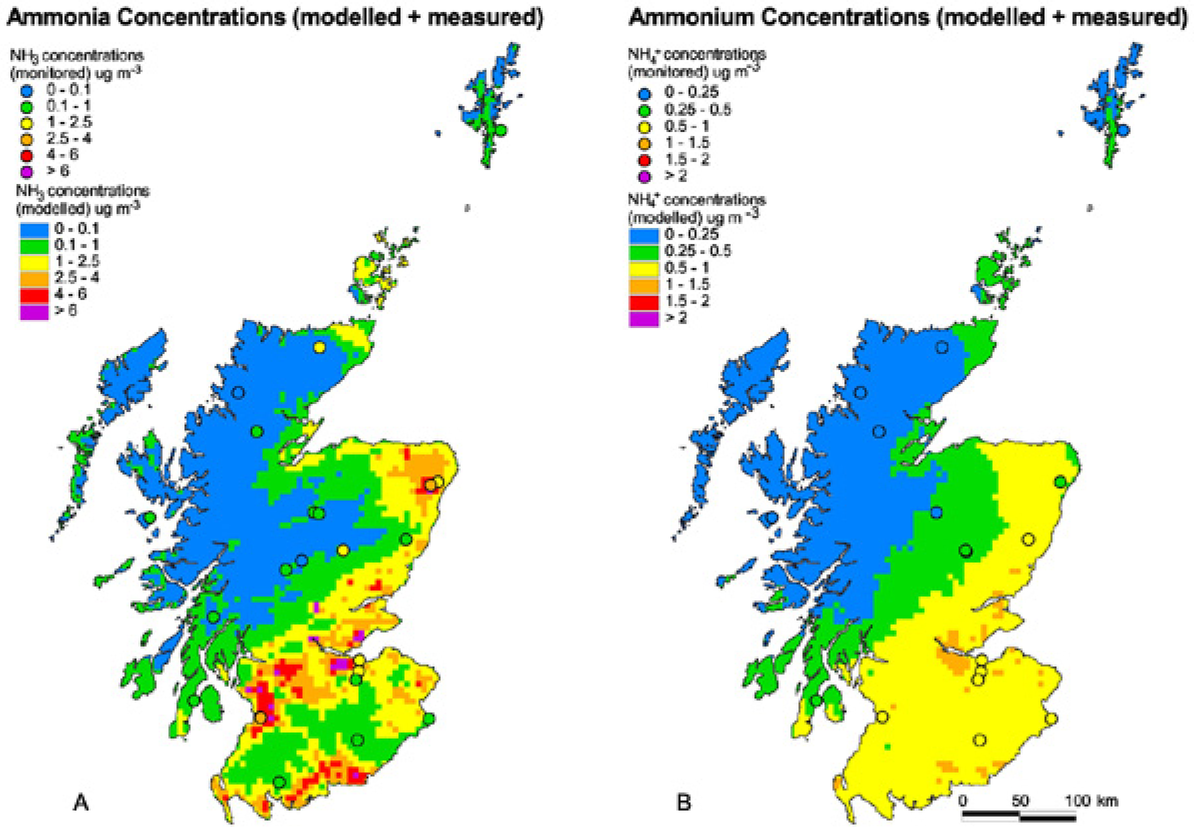

**FIGURE 6.** Spatial distribution of gaseous ammonia and aerosol ammonium across Scotland estimated by the FRAME model for 1996. The modelled values, shown on a 5-km grid, are compared with the mean measured concentrations (circles) from the National Ammonia Monitoring Network for the period 1996–1999.

years. Most of Scotland is dominated by $NH_3$ emissions from cattle. Conversely, sheep are estimated to be the main source in only a few areas of the Southern Uplands and southeast Highlands. Emissions from pig and poultry farming are rather localized, but explain the main “hot spots” of ammonia emission. Nonagricultural sources are more uncertain, but are estimated to dominate both in urban areas and in some low emission areas, due to seabird and wild animal emissions. Finally, it may be noted that nowhere in Scotland do crops provide the main $NH_3$ emission source, as sometimes occurs in eastern England[19]. However, some of the mixed areas in eastern Scotland include a significant contribution from this category.

## Ammonia Concentrations and Deposition

Estimates from the FRAME model for both gaseous $NH_3$ and aerosol $NH_4^+$ concentrations are shown in Fig. 6. Fig. 6 also shows the values reported by the National Ammonia Monitoring Network. The FRAME model estimates for $NH_3$ concentration vary by a large amount on a local level, reflecting the emission inventory and rapid dispersion away from ground-level sources. Conversely, the distribution of $NH_4^+$ concentrations modelled by FRAME is much smoother for two reasons: firstly, $NH_4^+$ is a secondary product that takes some time to form in the atmosphere and, secondly, $NH_4^+$ has a longer lifetime in the atmosphere than $NH_3$. As with $NH_3$, the lowest $NH_4^+$ concentrations are estimated by FRAME to occur in the most remote parts of northwest Scotland, where values are less than 0.2 $\mu g\ m^{-3}$.

The values from the National Ammonia Monitoring Network are shown as coloured dots in Fig. 6, overlaying the model estimates and support the very spatially variable values of $NH_3$ concentrations, but rather smooth spatial pattern of $NH_4^+$ aerosol. Both maps indicate an encouraging agreement between the model and measurements, which gives substantial support to the use of FRAME in mapping values across the country.

## Potential Impacts of Nitrogen Deposition

The deposition of $NH_x$ and $NO_y$ are estimated to have significant potential impacts across wide areas of Scotland. Based on the mapping of estimated critical loads and total atmospheric nitrogen deposition, around 75% of the 1-km squares containing coniferous and deciduous woodlands exceed the critical loads for nutrient nitrogen, while the areas exceeded for bogs and heathlands are 45 and 19%, respectively[26]. These values are illustrated in Fig. 8A which shows the exceedance of the nutrient nitrogen critical load for deciduous woodlands. Fig. 8 shows substantial exceedance of the critical load in all lowland areas of Scotland, as well as in the Southern Uplands. The most sensitive change that is likely to be seen in practice is a change in the diversity of the woodland ground flora. Dramatic changes in ground flora have been observed near intensive livestock farms in Scotland[8,9]. Over the much wider area where the critical load is shown here to be exceeded, the changes may be more subtle or take a longer time (several decades) to appear. Despite this concern, it is encouraging to note that impacts of nitrogen deposition are not expected in the Atlantic deciduous woodlands of the northwest.

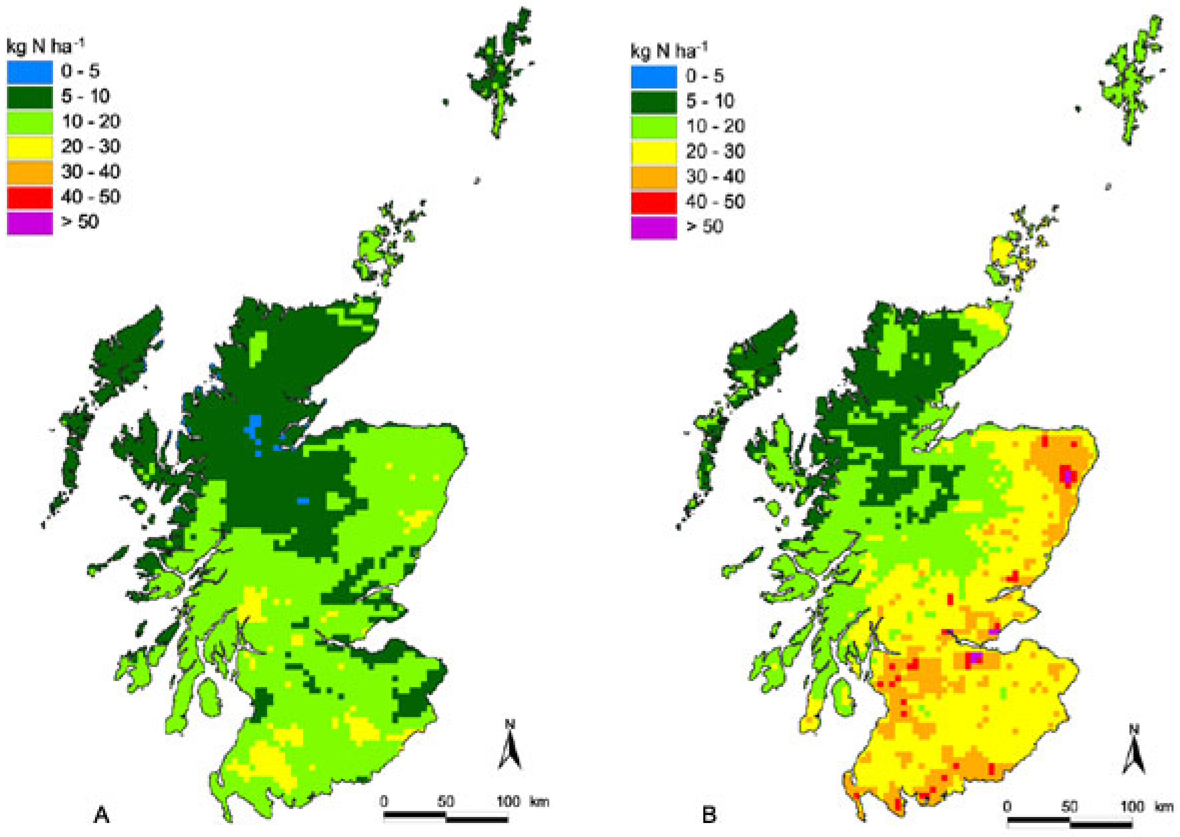

**FIGURE 7.** Modelled deposition of atmospheric nitrogen to Scotland, including both $NH_x$ and $NO_y$ for 1996. (A) Average total deposition accounting for the grid-average dry deposition to each 5-km grid square (i.e., net removal from the atmosphere). (B) Total dry deposition as received by woodlands and forest if present.

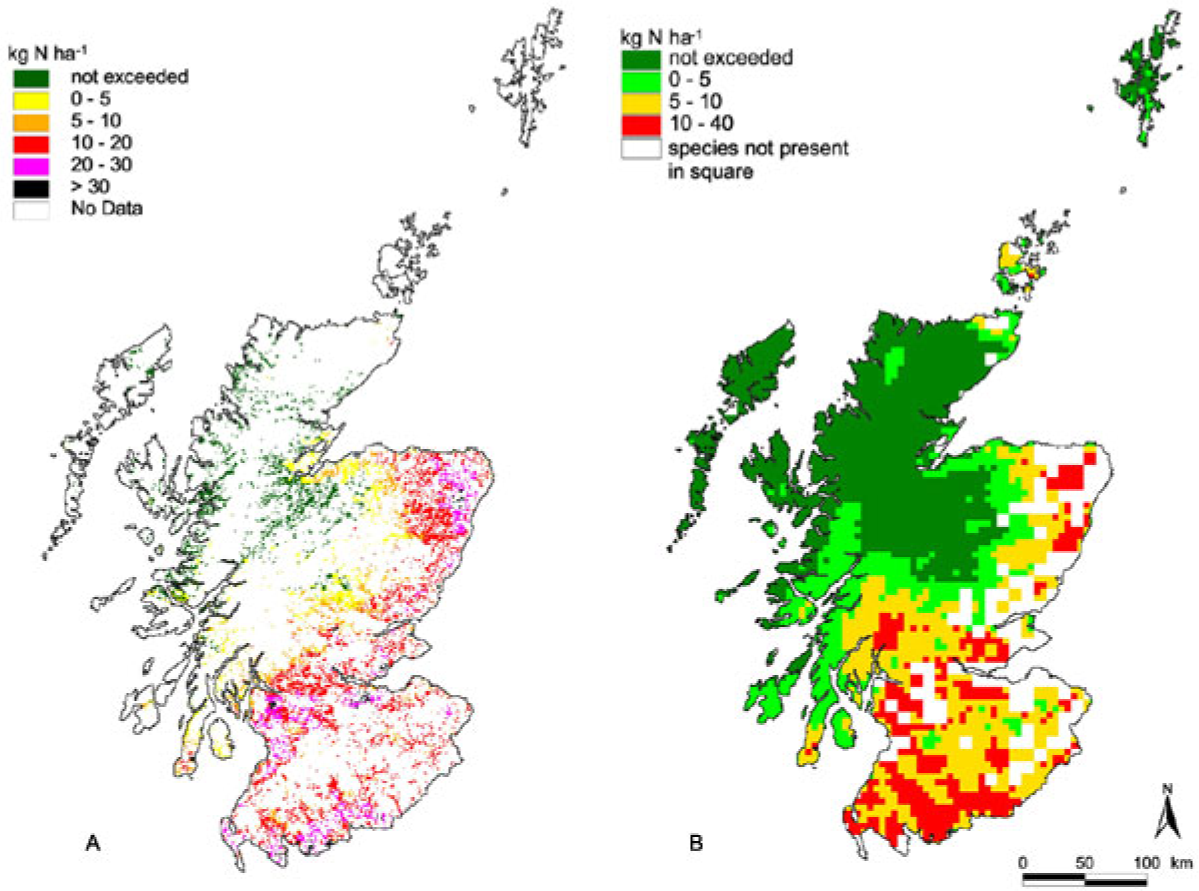

**FIGURE 8.** Modelled exceedance of the critical load for nitrogen eutrophication for (A) deciduous woodland and (B) sundew (*D. rotundifolia*). For coniferous woodland, a mass balance calculation is applied, while for *Drosera*, an empirical critical load of 10 kg N $ha^{-1}$ $year^{-1}$ has been set. For the deciduous woodland map, "no data" refers to areas with less than 5% cover of deciduous woodland.

Fig. 8B provides an example of critical load exceedance for nutrient nitrogen at a species level. Sundew is a key indicator species of acidic mire conditions, being naturally adapted to conditions of low nitrogen availability. Growth under low N availability is reflected in its insectivorous habit, designed to procure more nitrogen than is available from the soil. Sundew is, however, a rather slow-growing species that would be expected to be out-competed by faster growing species such as cotton-grass (*Eriophorum* spp.), where surplus nitrogen is available[27]. Fig. 8B indicates that the critical load for sundew is exceeded over most of Scotland, with the exception of the central and northwest Highlands. Fig. 8B suggests a threat to this species in central, eastern, and southwest Scotland.

## DISCUSSION

### Impacts and Ammonia Budgets

The above analysis demonstrates how a combination of modelling and measurement activities has been used to address the distribution of ammonia emissions, deposition, and impacts in Scotland. The maps produced are particularly useful to distinguish the areas that are either most or least affected, and to identify the key contributors. While the results shown here have focused on ammonia, it is clear that the eutrophication impacts illustrated are due to total nitrogen deposition. It is relevant to note, however, that deposition of $NO_y$ alone does not lead to critical load exceedance. While grid-averaged nitrogen

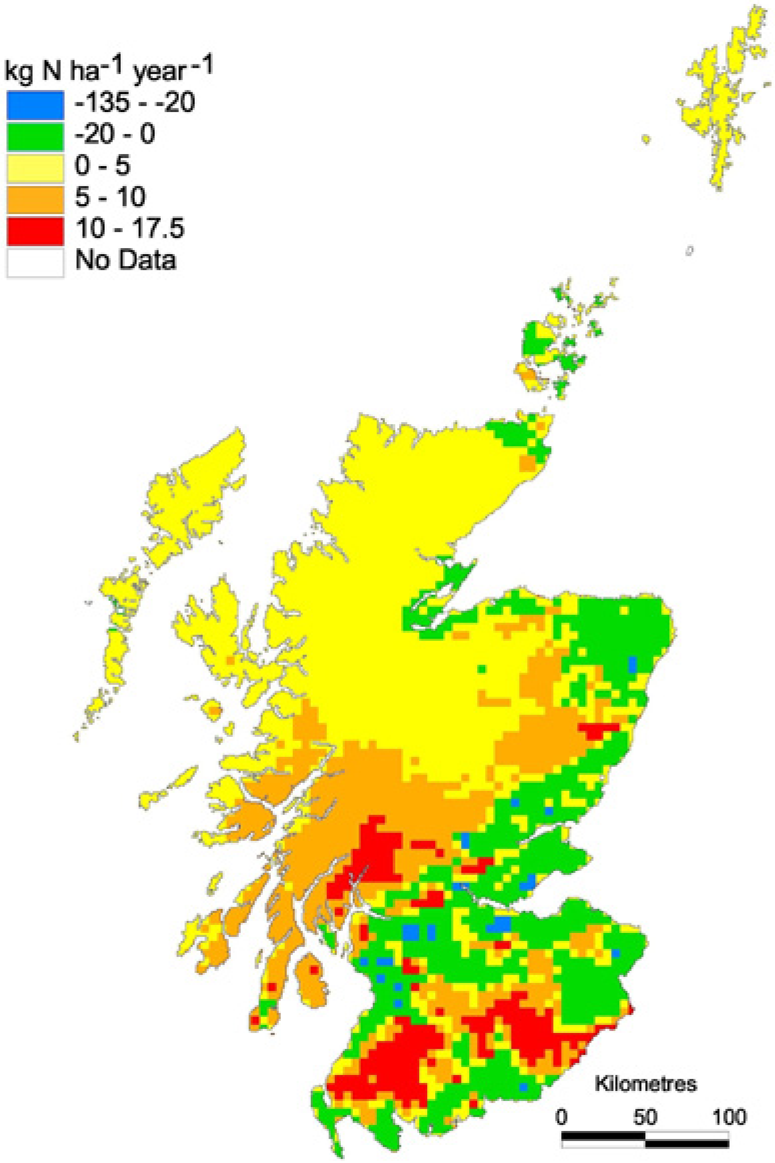

**FIGURE 9.** Modelled difference between $NH_3$ emission and grid-averaged $NH_x$ deposition in Scotland for 1996. In areas with positive values there is a net import of $NH_x$ (deposition larger than emission), while in areas with negative numbers there is a net export of $NH_x$ (emission larger than deposition).

deposition (Fig. 7A) of $NO_y$ and $NH_x$ are of similar magnitude, because of the high affinity of $NH_3$ for unfertilized land, the deposition received by the most sensitive habitat types (e.g., Fig 7B) reflects a much larger contribution from $NH_x$ than from $NO_y$. As a consequence, it is clear that efforts to abate nitrogen emissions should not focus on $NO_y$ alone, but must also address $NH_3$[1,5].

While the primary concerns noted here reflect the impacts of $NH_3$ deposition on sensitive habitats, $NH_3$ exchange may also be identified as a relevant term in estimates of nitrate leaching at a regional scale. This may be demonstrated by considering the net $NH_3$ budget of deposition minus emission on a 5-km grid basis. Fig. 9 shows that the lowland areas estimated to have the highest $NH_3$ emissions would have a significant net export to the atmosphere, with several areas estimated to have a net loss of more than 20 kg N $ha^{-1}$ $year^{-1}$. Conversely, the upland areas in the vicinity of sources receive a significant net gain of ammonia, with deposition being over 10 kg N $ha^{-1}$ $year^{-1}$ larger than the emission over wide areas of the Southern Uplands and southwest Highlands.

Budgets for $NH_3$ can also be considered in terms of the net import and export to the country. While the domain of FRAME is the whole of the British Isles, it may be divided into subdomains to consider regional import and export estimates. According to the FRAME estimates, England, Eire, and Northern Ireland are net exporters of $NH_x$, reflecting their large $NH_3$ emission and, in the case of Ireland, its position on the western edge of Europe. By contrast, Scotland can be considered "ammonia neutral", as it is estimated by FRAME to export a similar amount to that imported. This can be explained by the position of Scotland downwind (NE) of Northern Ireland, and the large area of uplands that act as net ammonia sinks (Fig. 9).

## Uncertainties

It must be recognized that there are substantial uncertainties in the estimates reported here. The emission estimates of the AENEID model include uncertainties in the total emission estimates[2,4], the distribution methodology[15], as well as in the underlying agricultural census and survey data. Similarly, the FRAME model includes many approximations, such as straight-line trajectories, estimation of U.K. import from a linked model TERN[28], and uncertainties in each of the chemistry, wet, and dry deposition parametrizations. The only way to test the performance of these models is by comparison with measurements of the National Ammonia Monitoring Network, as well as the wet-deposition monitoring network. The differences observed in Fig. 6 integrate the full range of uncertainties. In both cases, the agreement is encouraging, particularly for ammonium aerosol. The reason for this is likely to be the dependence of measured $NH_3$ concentrations on local sources, coupled with local uncertainties in the AENEID estimates, which tend to be averaged out for $NH_4^+$ aerosol due to its longer atmospheric lifetime. The uncertainties in the AENEID model are anticipated to be larger in lowland areas for pig and poultry farming, since it is difficult to model the spatial distribution of these $NH_3$ emissions according to land cover type, given the constraints in the detail of the available agricultural census data. For $NH_3$ emissions from cattle and sheep, the methodology appears to be much more robust. This has been tested in a local scale study in Glenshee (east central Highlands), where the emission estimates of the AENEID model were applied in the LADD model at a 250 m and a 500 m resolution, with encouraging agreement between measured and modelled $NH_3$ concentrations[29].

## National and Local Ammonia Abatement Policies

Recognizing the environmental impacts of ammonia, there are now a number of policy measures that are being established at an international level aimed at controlling ammonia emissions. These include a national target of annual $NH_3$ emissions of 297 kt $NH_3$ (or 244 kt $NH_3$-N) by 2010 under the Gothenburg Protocol of the UNECE Convention on Long Range Transboundary Air Pollution[30], as well as the EU National Emissions Ceilings Directive. In addition, the EU Directive on Integrated Pollution Prevention and Control (IPPC) now includes the application of Best Available Techniques (BAT) to reduce $NH_3$ emissions from large pig and poultry farms[31]. Each of these measures has some degree of equitability since they share the burden of emission control between the different countries of Europe or between the different sector industries that contribute to emissions. A caveat to this is that IPPC does not currently include measures for farm types other than intensive poultry and pigs. It must be recognized, however, that the proposed emission ceiling for 2010 is only a modest 11% reduction from the estimated emission for the protocol base year, while the agreed measures to define BAT are unlikely to reduce $NH_3$ emissions from the pig and poultry sectors by more than 10%. Given the extent of critical load exceedance revealed in Fig. 7 across Scotland, it is clear that a substantial ammonia problem will remain by 2010, even where there is full compliance with the existing policy measures. While further $NH_3$ abatement may be possible by implementing technical measures such as manure injection more widely, it is unlikely that this will exceed 20–30% abatement for the U.K.[32].

In looking for complementary solutions to the ammonia problem, it is important to recognize the high degree of spatial variability of ammonia deposition and critical load exceedance, as well as the local nature of much of the deposition. Where Scotland takes on board $NH_3$ emission controls, this is good news, as it means that the benefits will largely occur in Scotland. It also means that much of the critical load exceedance is due to $NH_3$ emissions in the vicinity. For example, Sutton et al.[33] found that on average 60% of the $NH_3$ dry deposited to 5-km grid squares was the result of emission within the same square. Even more local than this, it may be noted that within the rural landscape a nature reserve will be more at risk where it occurs in the immediate vicinity of an ammonia source. For example, Dragosits et al.[34] showed elevated exceedance up to ~1–2 km from a large intensive livestock farm or up to ~300 m from a small mixed farm and intensively managed fields. As a result, both the edges of nature reserves or small nature reserves within agricultural landscapes are much more at risk than the centres of large nature reserves or reserves remote from intensive agricultural activity. The 5-km maps shown here will both underestimate the extent of exceedance near to sources, and overestimate it where a receptor within a 5-km grid square is distant from the sources.

The consequence of these effects is that where a nature reserve is adjacent to farm $NH_3$ sources in the rural landscape, the typical levels of $NH_3$ abatement (e.g., 10%) will be inadequate to avoid critical load exceedance. By contrast, much can be gained in terms of reducing $NH_x$ deposition to sensitive receptors by maximizing the distances between the sources and sensitive areas such as nature reserves. This effect points to the need to further develop and utilize spatial planning approaches in the context of protecting sensitive areas from $NH_x$ deposition. Such approaches are already considered as a means to reduce urban pollution, e.g., planning considerations for locating new roads. In the case of new developments that emit $NH_3$, these developments should not be recommended in the vicinity of such priority sensitive ecosystems, and instead should be encouraged to be sited elsewhere. Similarly, more stringent $NH_3$ emission abatement measures would be justified (e.g., under IPPC) where an existing $NH_3$ source is located in the vicinity (e.g., < 2 km) of a sensitive area. In the case of farm $NH_3$ emissions, spatial measures need to be taken for both emissions at the farm (housing and manure storage emissions) and emissions at a field level (manure spreading, grazing, and mineral nitrogen fertilization). Such spatial measures might be considered less equitable in that they would not lead to a “level playing field” of ammonia abatement targets across the country. By contrast, they would allow efforts to be focused at the location of the problem, reducing the overall burden of abatement for a given level of environmental benefit.

## ACKNOWLEDGEMENTS

We are grateful for funding of this work from the Scottish Executive (subcontractor to ENTEC UK Ltd. under ZEC5/22), Scottish Natural Heritage (project BAT/NA02/99/00/097), and the Department of Environment, Food and Rural Affairs (Air and Environment Quality research programme). Fig. 9 was prepared following a useful discussion with A. Vinten of the Scottish Agricultural College.
